# Updating beliefs about pain following advice: Trustworthiness of social advice predicts pain expectations and experience

**DOI:** 10.1016/j.cognition.2024.105756

**Published:** 2024-03-04

**Authors:** Charlotte Krahé, Athanasios Koukoutsakis, Aikaterini Fotopoulou

**Affiliations:** a Department of Psychology, Institute of Psychiatry, Psychology, and Neuroscience, King’s College London, London, United Kingdom; b Research Department of Clinical, Educational and Health Psychology, University College London, London, United Kingdom; 1 Now at School of Psychology, Liverpool John Moores University, Liverpool, United Kingdom

**Keywords:** Pain, Social influence, Social context, Trustworthiness, Epistemic trust, Expectation, Belief updating

## Abstract

Prior expectations influence pain experience. These expectations, in turn, rely on prior pain experience, but they may also be socially influenced. Yet, most research has focused on self rather than social expectations about pain, and hardly any studies examined their combined effects on pain. Here, we adopted a Bayesian learning perspective to investigate how explicitly communicated social expectations (‘advice about pain tolerance’) affect own pain expectations, and ultimately pain tolerance, under varying conditions of social epistemic uncertainty (trustworthiness of the advice). *N* = 72 female participants took part in a coldpressor (cold water) task before (self-learning baseline) and after (socially-influenced learning) receiving advice about their likely pain tolerance from a confederate, the trustworthiness of whom was experimentally manipulated. We used path analysis to test the hypothesis that social advice from a highly trustworthy confederate would influence participants’ expectations about pain more than advice from a less trustworthy source, and that the degree of this social influence would in turn predict pain tolerance. We further used a simplified, Bayesian learning, computational approach for explicit belief updating to examine the role of latent parameters of precision optimisation in how participants subsequently changed their future pain expectations (prospective posterior beliefs) based on the combined effect of the confederate’s advice on their own pain expectations, and their own task experience. Results confirmed that participants adjusted their pain expectations towards the confederate’s advice more in the high- vs. low-trustworthiness condition, and this advice taking predicted their pain tolerance. Furthermore, the confederate’s trustworthiness influenced how participants weighted the confederate’s advice in relation to their own expectations and task experience in forming prospective posterior beliefs. When participants received advice from a less trustworthy confederate, their own sensory experience was weighted more highly than their socially-influenced prior expectations. Thus, explicit social advice appears to impact pain by influencing one’s own pain expectations, but low social trustworthiness leads to these expectations becoming more malleable to novel, sensory learning.

## 1 Introduction

Pain is a subjective experience shaped by a range of psychological processes and the context in which it is experienced (Krahé, Springer, Weinman, & Fotopoulou, 2013; Tracey & Mantyh, 2007; Wiech, 2016). In particular, prior expectations about pain are key in influencing pain experience (see e.g., Atlas & Wager, 2012; Bingel, 2020; Cormier, Lavigne, Choinière, & Rainville, 2016; Wiech, 2016). A wealth of research has focused on understanding the processes underlying ‘placebo’ (expectations of pain relief) and ‘nocebo’ (expectations of exacerbated pain) effects on pain (e.g., Bingel, 2020; Büchel, Geuter, Sprenger, & Eippert, 2014; Petrie & Rief, 2019) and their modulation by contextual factors (see e.g., Atlas, 2021). However, this research has mainly focused on the pain-modulatory role of our *own* expectations, rather than how *social* expectations influence pain.

From birth, we learn to regulate our bodily and emotional states through others (Fotopoulou & Tsakiris, 2017; Fotopoulou, von Mohr, & Krahé, 2022; Van Puyvelde, Collette, Gorissen, Pattyn, & McGlone, 2019; Winberg, 2005). For example, as infants cannot change their own temperature or assuage pain or hunger themselves, others act ‘on their behalf’ to change sensory input (Fotopoulou & Tsakiris, 2017). Hence, caregivers’ expectations and responses to infants’ physiological states presumably contribute to how infants learn about the regulation of such bodily states, including pain (Atzil, Gao, Fradkin, & Barrett, 2018; Fotopoulou & Tsakiris, 2017). Social variables and interactions modulate pain in infancy (Gennis & Pillai Riddell, 2018; Gursul et al., 2018), but also in adulthood (Sambo, Howard, Kopelman, Williams, & Fotopoulou, 2010; Hurter, Paloyelis, de Williams, & Fotopoulou, 2014; see Krahé et al., 2013, Che, Cash, Ng, Fitzgerald, & Fitzgibbon, 2018; Che, Robin, Sungwook, Paul, & Bernadette, 2018; Krahé & Fotopoulou, 2018, for reviews).

Yet, how and under which conditions *explicit social expectations*, e.g., *advice about pain*, influence pain remains incompletely understood. This is partly because existing research has focused on implicit social expectations, which are either not related or not directly communicated to the participant. For example, physicians’ pre-treatment expectations of pain relief affect patients’ pain outcomes (Galer, Schwartz, & Turner, 1997; Witt, Martins, Willich, & Schützler, 2012), and directly manipulating healthcare providers’ expectations regarding analgesic effects of (actually inert) creams influences participants’ pain in a simulated medical setting (Chen et al., 2019). However, social expectations were not communicated to the participant in such studies. Similarly, social observation paradigms show that our own pain can be influenced by what we observe in others, but what others may think about our potential for pain, and how we react to it, is not investigated. For example, studies have found that viewing someone simulating analgesic benefit enhances observers’ placebo responses (Colloca & Benedetti, 2009); that viewing others’ ratings of high pain increases own pain (Koban & Wager, 2016); and that being shown information that ‘typical’ participants have a high pain tolerance on coldpressor pain trials increases participants’ own pain tolerance (Pulvers, Schroeder, Limas, & Zhu, 2014). Such social- observation paradigms engage different brain networks to live social interactions, as demonstrated by a wealth of neuroscientific research contrasting these perspectives (see e.g., Redcay & Schilbach, 2019). Furthermore, social contexts without a ‘possibility for action’ impact pain to a lesser degree than social interactions with such a possibility (Krahé et al., 2013). Thus, critical questions remain: What happens when we receive direct social advice about our own pain on an upcoming task? How does this advice influence our own expectations and pain experience? And which factors influence whether we take on board this advice?

Accordingly, in the current paper, we adopted a Bayesian brain framework perspective to better understand the processes underlying effects of explicit social expectations on pain. Participants took part in a coldpressor (cold water) task before and after explicit social advice from a confederate about participants’ likely pain tolerance. We examined how participants changed their own pain expectations and then their actual pain tolerance based on the advice. We also investigated how participants subsequently changed their future pain expectations based on the combined effect of the advice on their pain expectations and their own experience. Furthermore, we experimentally manipulated the perceived trustworthiness of the person delivering the advice to investigate how varying uncertainty of social advice would impact the above measures. Finally, using a simplified, Bayesian learning, computational approach for explicit belief updating (Kirsch et al., 2021), we examined the role of latent parameters of precision optimisation in belief updating. We unpack the background of these experimental and computational aims and our specific hypotheses below.

Bayesian brain frameworks can offer a theoretical perspective and testable hypotheses for how explicit social expectations may influence pain, given that they have been used to describe the role of own expectations and wider context in shaping pain (e.g., Büchel et al., 2014; Kiverstein, Kirchhoff, & Thacker, 2022) and the social modulation of pain (Decety & Fotopoulou, 2015; Krahé et al., 2013; von Mohr & Fotopoulou, 2019), social conformity (Theriault, Young, & Barrett, 2021), and cultural learning more broadly (Veissière, Constant, Ramstead, Friston, & Kirmayer, 2020). Briefly, Bayesian brain frameworks put forward that prior learning is used to construct generative models about the hidden causes of sensory input, which are continually updated and improved with the aim of minimising uncertainty and maintaining homeostasis in a constantly changing world. Furthermore, combining this Bayesian perspective with a predictive coding scheme about sensory perception, uncertainty is minimised by reducing ‘prediction errors’ through two key processes: Under perceptual inference, prediction errors are reduced by updating expectations, based on prior beliefs, on the basis of sensory experience (see Strube, Rose, Fazeli, & Büchel, 2021). Under active inference (Friston, 2009, 2010), prediction errors are minimised by selectively sampling the environment (acting on the world or regulating one’s own states) to match one’s own prior beliefs about sensory experience. Moreover, in this framework, information-gathering actions can be called *epistemic actions*, as they aim to change one’s contextual beliefs (e.g., exploring new foods while travelling) and are distinguished from *pragmatic actions*, which aim to achieve goals (e. g., following a map to one’s destination; Friston et al., 2015).

In Bayesian, predictive coding models of pain, pain is thought to arise from the integration of expectations about pain experience, based on prior beliefs about pain, and incoming sensory information, such as nociceptive input. Importantly, the weighting of prior beliefs and incoming sensory (e.g., nociceptive) information in shaping pain depends on their relative uncertainty (mathematically, its inverse precision; Grahl, Onat, & Büchel, 2018) in different contexts and at different levels of the neurocognitive hierarchy. For example, at the level of perception, such accounts predict, and indeed a number of studies have shown (Brown, Seymour, Boyle, El-Deredy, & Jones, 2008; Colloca, Petrovic, Wager, Ingvar, & Benedetti, 2010; Hoskin et al., 2019), that experienced pain will be biased towards the expected level of pain, particularly when this expectation is of greater certainty (precision). This is because expectation certainty will lead to a suppression of bottom-up, nociceptive input, and greater importance being placed on the top-down, prior-related signals when forming posterior beliefs about pain. Likewise, the reduction of pain reports by placebo expectations (placebo analgesia) can be mapped well by formal Bayesian models (Anchisi & Zanon, 2015; Jung, Lee, Wallraven, & Chae, 2017). However, this perspective can be used to go beyond expectations based on the experimental cues given about the pain experience itself. Ample research in clinical settings has shown that cue-independent expectations, such as prior beliefs about one’s resilience (e.g., pessimistic or catastrophising beliefs about the consequences of a nociceptive stimulus on the body, such as excessive or permanent tissue damage, or on one’s mental wellbeing, such as fears of not being able to cope with pain; Hoskin et al., 2019), or the social environment where pain occurs (e.g., an environment where others may offer active support, see Krahé et al., 2013; Krahé, Drabek, Paloyelis, & Fotopoulou, 2016; von Mohr, Krahé, Beck, & Fotopoulou, 2018), can also have important effects on pain. To give a simple example, when faced with the nociceptive signals arising from a sharp object entering the skin (e.g., a syringe), the degree to which nociceptive signals will induce a pain experience will depend not only on prior beliefs about how painful injections can be, but also on expectations about the likely tissue damage an injection can cause, the likely benefits of such an injection, the individual’s ability to cope with the pain, and the social support the individual is likely to receive.

In this sense, the precision of prior beliefs versus incoming sensory input is critical for belief updating, by determining how prediction errors are weighted in the formation of posterior (updated) beliefs. Moreover, this updating also depends on how certain one is about the relevance or suitability of one’s prior beliefs about new sensory data in a given context. To return to the travelling example, for instance, one may feel that the precision of one’s prior beliefs about food options in a new country is rather low, so one needs to perform more epistemic actions (e. g., ask questions and advice from others) before updating one’s posterior beliefs and concluding on a course of action (e.g., ordering a meal). Following the received advice can, in this instance, be seen as reducing epistemic uncertainty based on explicit social expectations. The assumption further is that when one is then sampling the selected food, expectations about how the food will taste (based on social advice) will influence perception, given their perceived reliability in this otherwise uncertain context. Thus, it is assumed that in this form of cultural learning, people achieve a sort of biased perception of new experiences via expectations based on social advice taking. Generally, some theoretical models have extended the above active inference model to social influence, putting forward that to make one’s environment predictable and minimise expected uncertainty, which can be computationally costly and pragmatically risky, human agents calibrate their prior expectations based on social and cultural epistemic learning, not just sensory learning (Fotopoulou et al., 2022; Veissière et al., 2020). Thus, humans may have intrisic motivations to co-regulate their states with others (Atzil et al., 2018; Fotopoulou et al., 2022; Fotopoulou & Tsakiris, 2017) and to adjust to others’ expectations (Theriault et al., 2021), or more broadly to learn about their biological, physical and social enviroment based on how their own cultural environment dictates the reliability (i.e., precision) of certain expectations over others (Krahé et al., 2013; Veissière et al., 2020; von Mohr & Fotopoulou, 2019). Such a process could explain social observational learning findings, but has not yet been formally or experimentally examined in relation to explicit social expectations (advice) from others. Based on this perspective, we predict that participants would take on board social advice about pain depending on its expected epistemic value (formally, expected precision, and in psychological terms, reliability, and as we will see below, trustworthiness).

Not all social sources of information are equally relevant or indeed reliable in reducing epistemic uncertainty. In a social context, the ‘epistemic trust’ placed in social information critically impacts how it influences learning (Hertz, Bell, & Raihani, 2021). Epistemic trust refers to an individual’s willingness to consider new information as trustworthy, reliable, and salient (having high epistemic value; Albarracin, Demekas, Ramstead, & Heins, 2022). Epistemic trust can be examined at an individual level (e.g., by measuring individual differences in paranoia, as in Hertz et al., 2021), or the epistemic trustworthiness of information or indeed of social interaction partners can be experimentally manipulated. For example, experimentally-elicited perceived trustworthiness determines whether a person is evaluated as low or highly threatening (Oosterhof & Todorov, 2008), whether a person is approached or avoided (Slepian, Young, Rule, Weisbuch, & Ambady, 2012), and whether or not a person is trusted (Van’t Wout & Sanfey, 2008). Crucially, epistemic trust(worthiness) is thought to determine how much social information is deemed worth integrating into own expectations; in short, higher epistemic trust(worthiness) should confer greater subjective certainty, in the sense of reducing epistemic ambiguity, about social expectations, and thus increase their weighting in shaping one’s own expectations relative to new sensory evidence. Indeed, in relation to pain, trustworthiness facets such as warmth and competence (see Colquitt, Scott, & LePine, 2007) influence pain expectations (see e.g., Blasini, Peiris, Wright, & Colloca, 2018; Howe, Goyer, & Crum, 2017; Necka, Amir, Dildine, & Atlas, 2021) and pain (Campbell, Holder, & France, 2006).

In Bayesian brain frameworks, highly trustworthy social agents ‘wield epistemic authority’ (Veissière et al., 2020), that is, they confer greater precision (reducing epistemic ambiguity) on beliefs and experiences. In a vicarious pain observation paradigm, both the predictability (mean) and the reliability (standard deviation) of the vicarious pain ratings influenced participants’ pain responses (Yoshida, Seymour, Koltzenburg, & Dolan, 2013). However, such effects have not been examined regarding explicit social advice about upcoming pain. In the present study, we thus experimentally varied, between participants, the perceived epistemic trustworthiness of a person giving explicit social advice about pain tolerance prior to an upcoming coldpressor (cold water) task by presenting fake social information, ostensibly from previous participants, about this person’s level of trustworthiness (high vs. low). This person, a confederate, was initially present during a baseline coldpressor trial, and then provided participants with a prospective social expectation (advice) regarding their expected pain tolerance on the second trial. This combination of between- and within-subject manipulations allowed us to establish how much social advice influenced participants’ anticipatory pain beliefs and pain tolerance (and their relation) relative to their existing beliefs based on a previous trial where they had not had any explicit advice. We expected social advice from an epistemically trustworthy person to influence both anticipatory beliefs about pain and pain tolerance more than advice from a less trustworthy source. Specifically, we hypothesised that in this explicit ‘social advice’ setting, participants would adjust their expectations towards the expressed advice more when provided by a confederate appearing high (vs. low) in trustworthiness (Hypothesis 1). Further, we extended studies indicating that social expectations that are either not related or not directly communicated to the participant affect participants’ pain (see above) to examine how explicitly communicated social advice, and critically, the degree to which it influenced participants’ own expectations, would shape pain tolerance. The confederate in this study gave participants positive advice (that they could tolerate the coldpressor longer than they themselves thought; see Methods below). Therefore, we hypothesised that greater social advice taking would predict greater pain tolerance (Hypothesis 2). We tested these hypotheses using path modelling, entering our variables in the temporal order in which they were measured to assess the impact of perceived trustworthiness and social advice taking on pain.

Furthermore, we conducted separate analyses based on a different computational modelling approach best suited to our belief-updating task and framework to examine the combined influence of social advice and pain experience on participants’ future pain expectations (posterior prospective beliefs sampled following the second coldpressor trial). Although the high ecological validity of both our coldpressor task and the ‘live’ social interaction setup did not allow sufficient trials for full computational modelling, we used a simplified, Bayesian learning, computational approach for explicit belief updating (Kirsch et al., 2021) to investigate the role of latent parameters of precision optimisation in belief updating. Specifically, we used different experimental measurements of both social and sensory uncertainty as sources for prior belief or evidence precision to build five different Bayesian belief-updating models (including two baseline models) and examined which model best approximated participants’ explicitly reported future pain expectations (prospective posterior beliefs). We examined model fit in terms of how generated posterior beliefs corresponded with participants’ actual posterior beliefs, and compared learning rates between trustworthiness conditions to see whether participants showed greater new learning in the low- vs. high-trustworthiness condition. We expected that receiving advice from a high- vs. a low-trustworthy confederate would lead to reduced subsequent learning based on new sensory evidence, given that participants would have greater subjective certainty in their priors influenced by social advice from a highly trustworthy vs. less trustworthy person relative to new sensory information. By contrast, social advice from a less trustworthy person was predicted to render prior beliefs more uncertain and hence malleable to new sensory evidence (Hypothesis 3).

In summary, we predicted that social advice given by a trustworthy source would be judged as more reliable (than that of a less trustworthy source) and hence would also lead to expectations about pain held with greater subjective confidence. This, in turn, would lead to the subsequent suppression of bottom-up, nociceptive input relative to the top-down, prior-related signals when forming posterior beliefs about pain. Thus, we envisioned a relationship between the perceived reliability, or trustworthiness, of social advice and the subjective confidence by which one holds one’s own priors, even when these beliefs are about interoceptive signals originating in one’s own body. Confirmation of these hypotheses would imply that trustworthy social sources can influence not only how we experience the external world, but also signals originating in our own body.

## 2 Methods

### 2.1 Design

**Fig. 1:**
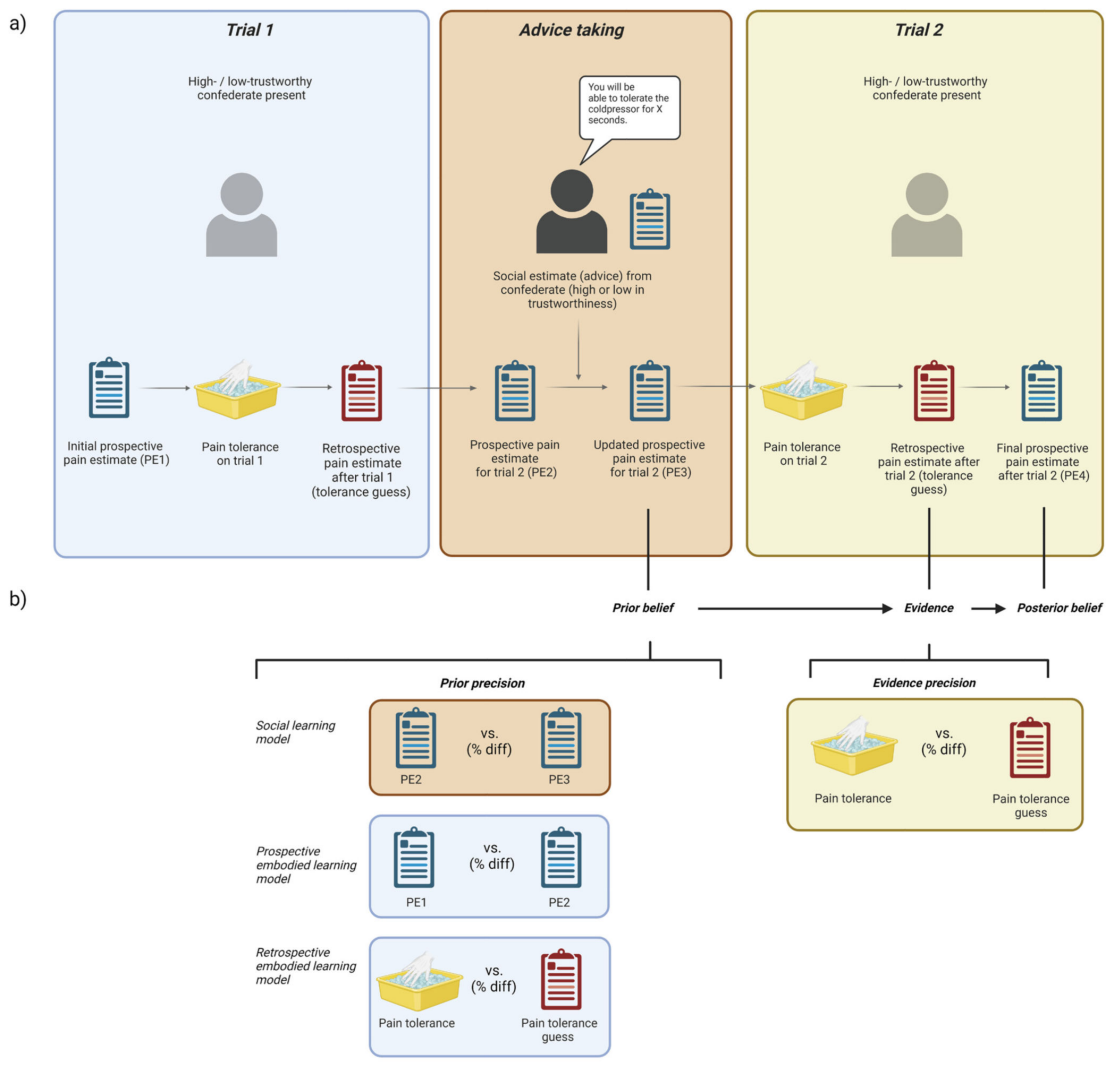
A schematic representation of the study design and procedure depicting (a) the coldpressor trials, social conditions, and main outcome measures, and (b) measurements of social and sensory uncertainty used as sources of prior precision and evidence precision in our Bayesian belief updating models. (For interpretation of the references to colour in this figure legend, the reader is referred to the web version of this article. This figure and Figures 3 and 4 were created with web link Bio-Render.com)

We studied the influence of explicitly-communicated social advice regarding participants’ pain tolerance and the perceived epistemic trustworthiness of the person giving advice on pain tolerance and own beliefs about pain tolerance in the context of painful coldpressor trials. We manipulated perceived epistemic trustworthiness (high, low) of a research confederate present during the experiment in a between-subjects manner by means of fake feedback from previous participants. Participants then completed two coldpressor trials in the presence of the high-/low-trustworthy confederate; see Fig. 1 (panel a) for a schematic outline of the procedure. Trial 1 was a ‘baseline’ trial; a baseline or reference experience is often needed to induce expectation effects in healthy, pain-free individuals (Chen et al., 2019). During this trial, the confederate was present, but did not provide advice. Then, before the second trial, the confederate provided advice specifically about how long they thought the participant would be able to tolerate the cold water on the main trial; this was a positive expectation, that is, the confederate always thought participants could tolerate the coldpressor for longer than they thought themselves. We made this pragmatic decision because we reasoned that negative expectations, that is, advising on low pain tolerance, might lead all participants to swiftly withdraw their hands from the water irrespective of trustworthiness condition, simply to terminate the task earlier (a socially-encouraged ‘out’). Participants then took part in the second, main trial. Outcomes of interest on each trial were participants’ prospective and retrospective estimates of pain tolerance (cognitive expectations regarding how long participants thought they would tolerate the cold water before and after each trial, and how long they guessed they had tolerated it on each trial), and pain tolerance (behavioural measure; point at which the hand was withdrawn from cold water). Pain tolerance was the primary outcome measure for pain experience. Furthermore, we measured pain threshold (behavioural measure i.e., the point at which pain was first reported), and pain intensity (reported on visual analogue scales) during each trial and included these as covariates in the analyses, alongside age and hand temperature (see *Plan of Analysis*).

### 2.2 Participants

Eighty-one female participants were recruited to take part in this study. As interaction effects between participant sex and experimenter sex have been noted (pain report is influenced by whether the experimenter is of the same or opposite sex; Kállai, Barke, & Voss, 2004), and as the research confederate was female, we held sex constant and recruited only female participants (see Discussion for details and limitations of this choice). We included participants who were fluent in English, did not have Raynaud’s syndrome or chronic pain, and did not have a history of psychiatric or neurological problems, drug abuse, or known cognitive impairment. Of the 81 participants, two were excluded because it transpired during the testing session that they did have chronic pain, four were excluded because they indicated at debriefing that they had not believed the trustworthiness manipulation, and three were excluded because they did not follow instructions during the pain paradigm. The final sample thus consisted of *N* = 72 women (mean age: 23.86 years, *SD* = 6.27), of whom *n* = 34 were in the low- and *n* = 38 in the high-trustworthiness condition. Ethical approval was obtained from King’s College London Psychiatry, Nursing and Midwifery Research Ethics Subcommittee.

### 2.3 Procedure

Participants were invited to take part in a study on the role of others in the experience of pain. Upon arrival, participants met an experimenter and were given a cover story, stating that the purpose of the experiment was to examine how a medical student’s perception of pain related to participants’ perception of pain; the department in which the study was conducted was responsible for training medical students in psychology. Participants were told that they would take part in a coldpressor trial and that a medical student (the confederate), who was keen to improve how well she could estimate participants’ pain, would join the experiment before the first coldpressor trial. They were further informed that after watching the initial coldpressor trial, and before the second, main trial, the medical student would provide an estimate about participants’ pain tolerance. Under the guise of the medical student (confederate) aiming to improve her performance, participants were then shown feedback from previous participants about how they had found the student. This feedback comprised high or low trustworthiness statements (depending on condition), presented in a PowerPoint presentation which participants viewed at their own pace, and after which they filled in manipulation checks. Subsequently, the confederate entered the room and was introduced to participants as the medical student who would be giving the estimate and be responsible for monitoring the coldpressor task. Of note, the confederate was the same person in the high- and low-trustworthiness conditions.

Before the start of trial 1, participants submerged their hand in lukewarm water until their hand temperature fell between 28 and 32 °C to standardise hand temperature before the onset of the trial (see Mitchell, MacDonald, & Brodie, 2004). Then, participants gave an initial estimate regarding how long (in seconds) they thought they would be able to tolerate the cold water (initial prospective pain estimate or PE1; see Fig. 1). Importantly, participants were not told the maximum length of the trials (similar to Pulvers et al., 2014), that is, that trials would be terminated after 180 s (see below).

Specifically, they were instructed:

“Now, before we start, I’m going to ask you to estimate your pain tolerance. By pain tolerance, we mean how long you can stand the task, that is, how long you can keep your hand in the water until it becomes unbearable.”

Participants wrote these down out of sight of the experimenter and confederate. Then, they took part in the first coldpressor trial. Participants submerged their hand (hands were alternated between trials and hand order was counterbalanced across participants) in the water and were instructed to leave their hand in the water until the sensations became too uncomfortable or until told to remove their hand (after 180 s). They were instructed to indicate when they first felt pain, and the time was noted as the pain threshold. Furthermore, every 20s, participants were asked to rate their pain intensity on scales mounted on a stand out of sight of experimenter and confederate. During the trial, the confederate monitored water temperature by means of thermometer, ostensibly to secure the safety of the participant in line with the cover story and to enhance the relevance of the trustworthiness manipulation. The trial ended once participants withdrew their hand or after 180 s, whichever was sooner. Pain tolerance (length of time the hand was submerged) was recorded. At the end of the trial, participants gave a final pain intensity rating and were asked how long they thought they had kept their hand in the water (retrospective pain estimate i.e., tolerance guess). Then, they were asked to provide a prospective pain estimate for the next trial (PE2):

“Great, now that you’ve done that and before we do the next trial, could I ask you to write down how long you think you will be able to tolerate the coldpressor task using your other hand?”

Then participants completed the demographic questions, during which time the confederate computed the value of their estimate (advice; see below) and then verbally informed participants of their estimate:

“Alice [name of confederate] has estimated that you will be able to tolerate the coldpressor for X seconds, or X minutes, in this next trial.”

Then, before submerging their hand in the water for the main trial, participants provided their updated pain estimate for trial 2 (PE3).

“So, having done the coldpressor once yourself and having heard Alice’s estimation, I’m going to give you the chance to estimate once more – silently, in writing – how long you will be able to tolerate the coldpressor for on this task.”

Participants then completed trial 2 (other hand) while the confederate was again present. After trial 2, participants again gave a retrospective estimate (tolerance guess) and provided their final prospective pain estimate (PE4):

“This is the last estimate I’ll ask you for, but could you please imagine that you’re doing the experiment again. How long do you think you could tolerate the coldpressor for next time?”

Finally, participants filled in a sham feedback form about the confederate for future participants (to maintain the cover story), were fully debriefed, and paid £10 for their time.

### 2.4 Materials and measures

#### 2.4.1 Coldpressor apparatus

The coldpressor apparatus consisted of a customised insulated water container (64.5 × 37.5 × 36.5 cm), which was split into two compartments by means of a perforated plastic divider to allow water flow from one compartment to the other. One compartment was filled with blocks of ice. The other compartment was free of ice and fitted with a perforated plastic arm rest to enable participants to comfortably submerge their hand and wrist in the water (see also Hurter et al., 2014). During the trials, the confederate circulated the water out of reach of the participant’s hand with a metal instrument to avoid a buildup of warmer pockets of water around the participant’s hand in the coldpressor apparatus (see von Baeyer, Piira, Chambers, Trapanotto, & Zeltzer, 2005, for the importance of cold water circulation on coldpressor tasks). The average water temperature was *M* = 0.81 °C (*SD* = 0.84) on trial 1 and *M* = 0.77 °C (*SD* = 0.67) on trial 2 (comparable to previous studies; Mitchell et al., 2004; as trials were not directly compared, differences across trials were not taken into account in analyses). Participants were instructed to leave their hand in the water until the sensations became too uncomfortable or until told to remove their hand (after 180 s). Time was recorded from the moment participants submerged their hand in the coldpressor apparatus.

#### 2.4.2 Pain experience

##### 2.4.2.1 Pain tolerance

Pain tolerance was the primary outcome measure of pain experience in our frequentist analysis and was included in computing evidence precision in Bayesian analyses. Time was recorded from the point of submersion (0 s) to a maximum of 180 s. The coldpressor trial was always terminated after 180 s, as recommended by previous research (von Baeyer et al., 2005). The time (seconds from onset of trial) at which participants withdrew their hand was recorded as their pain tolerance on both trials.

##### 2.4.2.2 Pain threshold and pain intensity ratings

Pain threshold and pain intensity ratings were included as covariates in our path analysis. Pain threshold was defined as the time point (measured in seconds from onset of the trial) at which participants indicated that they started experiencing pain and was recorded on both trials. Pain intensity ratings were collected on both trials using horizontal visual analogue scales (VAS) with the anchors 0 (*no pain*) to 100 (*pain as bad as it could be*) every 20 s from onset of the trial until they withdrew their hand or the trial terminated at 180 s. Participants’ initial and final pain intensity rating were included as covariates in analyses.

#### 2.4.3 Own and social pain expectations

##### 2.4.3.1 Participant pain tolerance estimates

To obtain cognitive expectations about pain tolerance, participants were asked to prospectively estimate (in seconds) the length of time they thought they would be able to keep their hand submerged in the coldpressor apparatus (see above). Participants provided four such estimates: initial prospective pain estimate before trial 1 (PE1), prospective pain estimate for trial 2 (PE2), updated prospective pain estimate for trial 2 following the social expectation (PE3), and a final prospective pain estimate after trial 2 (PE4). In addition to these prospective expectations, participants also estimated, retrospectively, how long (in seconds) they thought they had tolerated the coldpressor on the previous trial (pain tolerance guess; see Fig. 1) before giving their prospective estimates for the next trial. Estimates were written on a sheet of paper mounted on a stand, which was obscured from the experimenter and confederate’s view. Prospective estimates were used in computing advice taking (see below) and in computing priors, posteriors, and precision of prior beliefs in Bayesian analyses. Pain tolerance guess on trial 2 was included as evidence and evidence precision in Bayesian analyses.

##### 2.4.3.2 Social advice

The confederate gave participants explicit, personalised advice regarding their predicted pain tolerance on trial 2 in the form of a prospective estimate. The estimate was set to be 35%, 50% or 65% (randomised across participants) higher than participants’ estimate at the end of the baseline coldpressor trial. We varied the % increase so that we were not simply measuring the discrepancy between fixed social expectation and participant expectations, but rather to examine effects across different levels of disparity between self and social expectations. As participants were not informed of the length of the trials, participants who kept their hands in the water for the full 180 s on trial 1 were still given higher estimates for the next trial. However, in that case, trial 2 was still terminated at 180 s. The three levels of % increase did not have an effect on how much participants changed their expectations, either alone or in interaction with trustworthiness condition (effect of % increase, *F*(2, 57) = 0.95, *p* = .392; effects of trustworthiness condition, *F*(1, 57) = 2.36, *p* = .130; interaction increase by trustworthiness condition, *F*(2, 57) = 0.31, *p* = .738). Thus, we collapsed across levels of increase in the analyses.

##### 2.4.3.3 Advice taking

To calculate how much participants took the advice of the confederate, that is, adjusted their own expectations following a social expectation, we adapted a formula devised for advice taking (Harvey & Fischer, 1997) and previously adapted by others (e.g., Miosga, Schultze, Schulz-Hardt, & Rakoczy, 2020). This formula yields the weight given to advice as a percentage. In the present study, a score of 100% meant that the participant fully incorporated the confederate’s advice, while 50% meant they equally weighted their own and the social expectation, and 0% meant they did not take advice into account at all.

The formula was as follows:

$$Advice\;taking=\frac{PE3-PE2}{Confederate\;estimate-PE2}\times 100$$

#### 2.4.4 Trustworthiness manipulation

Perceived trustworthiness (high, low) was manipulated using feedback (ostensibly from previous participants) about the trustworthiness of the research confederate. Feedback was presented as statements which were designed to capture the key dimensions associated with trustworthiness in previous research, namely ‘ability’ (competence), ‘benevolence’ (prosocial inclination), and ‘integrity’ (endorsing moral principles of fairness) facets of trustworthiness (Burke, Sims, Lazzara, & Salas, 2007; Colquitt et al., 2007). We constructed 18 statements (3 statements × 3 trustworthiness facets × 2 trustworthiness conditions) and piloted these in *N* = 19 participants (8 male, 11 female; mean age: *M* = 28.95 years, *SD* = 11.44). Pilot participants rated how trustworthy the confederate appeared to be in each statement using a scale from 1 (*not at all trustworthy*) to 7 (*extremely trustworthy*). The four highest-ranked high trustworthiness statements and four lowest-ranked low trustworthiness statements were selected for the high- and low-trustworthiness condition, respectively. These statements all related to the ‘benevolence’ and ‘integrity’ facets, as the ‘ability’ facet did not seem to capture trustworthiness as successfully. Statements for the high-trustworthiness condition included, “*I got the impression very quickly that Alice was a caring person and keen to estimate my pain correctly and safely*.” (benevolence) and, “*I noticed that Alice stayed focused and attentive to my pain throughout the experiment*.” (integrity). Statements for the low-trustworthiness condition included, “*I didn’t find Alice particularly caring and wasn’t sure whether her estimate was safe*.” (benevolence) and “*Alice didn’t seem that interested in becoming good at estimating others’ pain. It’s a shame because it could help her be a good and caring doctor*.” (integrity; see the cover story below). The complete set of statements for the high- and low-trustworthiness conditions are presented in Supplementary Materials, along with the trustworthiness facets and means and standard deviations for the pilot ratings.

To examine the success of the trustworthiness manipulation, participants responded to four statements regarding how much the confederate “seemed…caring / not keen to become a good doctor* / trustworthy / not a nice person*”, on a scale from 0 (*not at all*) to 7 (*extremely*). Statements marked by asterisks were reverse-scored, and an average across the four statements was created. Participants’ average ratings of the confederate for the low- and high-trustworthiness conditions were compared using a two-sample *t*-test to check that perceived trustworthiness conditions differed as intended (manipulation check).

### 2.5 Plan of statistical analyses

We investigated the influence of social advice and the perceived trustworthiness of the person giving the advice on (1) how participants changed their own pain expectations and then their actual pain tolerance based on the advice, and (2) how participants subsequently changed their future pain expectations (prospective posterior beliefs) based on the combined effect of a) the advice on their pain expectations and b) their experience. Accordingly, we first examined effects of advice taking on pain tolerance, and how both were influenced by trustworthiness condition. We then studied how participants updated beliefs under advice taking in the different trustworthiness conditions. Hypothesis testing focused on the main trial (trial 2), completed following the advice, but information from trial 1 was included in the belief-updating analyses (see Fig. 1).

#### 2.5.1 Influence of perceived trustworthiness on advice taking and pain tolerance

First, we examined effects of advice taking on pain tolerance and the influence of trustworthiness condition on these outcomes. To test Hypotheses 1 and 2, namely that trustworthiness condition would predict advice taking (greater advice taking in the high- vs. low-trustworthiness condition), which would in turn influence pain tolerance, we implemented path modelling in Mplus 8.0 (Muthén & Muthén, 1998-2017). Path analysis, a structural equation modelling technique, has several advantages over individual analyses to test these predictions: As our variables were assumed to be theoretically interconnected, their relationships were presumed not to be independent. In path analysis, the relationships between variables can be estimated simultaneously, accounting for variance explained in both outcomes. Trustworthiness condition (high, low) was dummy-coded (low = 0, high = 1). We estimated direct paths from trustworthiness condition to advice taking and from advice taking to pain tolerance, and additionally the indirect path from trustworthiness condition to pain tolerance via advice taking to assess any mediation effects. Age and hand temperature were included as covariates in the path from trustworthiness condition to advice taking, as they varied by trustworthiness condition. Pain threshold, and first and final pain intensity ratings were included as covariates for the path from advice taking to pain tolerance, as they were correlated with pain tolerance. As the pain tolerance outcome variable was skewed, we implemented bootstrapping (10,000 replications). The fit of the resulting over-identified model was evaluated using the chi-square test of model fit, root mean square error of approximation (RMSEA), comparative fit index (CFI), and standardised root mean square residual (SRMR). The model was judged to fit the data well if the chi-square test was non-significant, the RMSEA <0.06 to 0.08, CFI ≥ 0.95, and SRMR ≤0.08 (Schreiber, Nora, Stage, Barlow, & King, 2006).

#### 2.5.2 Computational approximation of belief updating under advice taking during conditions varying in epistemic trustworthiness

Next, we focused on participants’ belief updating after the main trial (trial 2). Using a simplified, Bayesian learning, computational approach for explicit belief updating (Kirsch et al., 2021), we sought to describe how participants would form posterior prospective beliefs about pain tolerance based on learning from sensory evidence (weighted by its precision, or reliability) and given participants precision-weighted prior beliefs. Specifically, the precision-weighted Bayesian posterior belief *μ*_*ϑ*/*y*_, was computed as follows:

$$\mu_{\vartheta /y}=\mu_{\vartheta }+\frac{{\pi_{\varepsilon }}^{*}s}{{\pi_{\varepsilon }}^{*}s+\pi_{\vartheta }}\left(y-\mu_{\vartheta }\right)$$

where *μ*_*ϑ*_ is the prior prospective beliefs about pain tolerance; *y* is the sensory evidence; *π*_*ε*_ is the precision of evidence; *π*_*ϑ*_ is the precision of the prior beliefs; and *s* is a scale factor (free parameter) we added to the model of Kirsch et al. (2021) to account for the potential scaling required to transform the *π*_*ε*_ proxy to the scale of the *π*_*ϑ*_ proxy (explained further below).

As stated above, we did not have sufficient trials to calculate precisions from updating patterns in the data themselves, but we had various explicit approximate measures of subjective uncertainty that we used as a proxy for these precisions. Using these different experimental measures of both social and sensory uncertainty as sources for prior belief or evidence precision (*π*_*ϑ*_ and *π*_*ε*_respectively), we built five different Bayesian belief-updating models and examined (using the Bayesian Information Criterion) which model best predicted participants’ explicitly-reported, future pain expectations (prospective posterior belief *μ*_*ϑ*/*y*_). In all five models, *prior belief (μ*_*ϑ*_*)* and *evidence (y)* were held constant. In the three non-baseline models, *evidence precision (π*_*ε*_*)*, was held constant and the *precision of the prior belief* (*π*_*ϑ*_) was varied (see Fig. 1, panel b, and Supplementary Materials). The scale factor *s* was applied on *π*_*ε*_ because, considering we are using precision proxies, we needed the models to capture how the relative changes of each of *π*_*ε*_ *and π*_*ϑ*_ reflected participants’ belief updating, rather than these proxies’ absolute levels. We calculated one global, across-participants value of *s* per condition and model as we did not have the number of trials required to compute one value per participant. *Prior belief* was participants’ anticipatory belief about pain tolerance in the 2nd trial, following the initial trial and social advice. As *evidence*, we specified participants’ retrospective estimate (tolerance guess), weighted by the difference between participants’ perceived and actual pain tolerance (*evidence precision*). This gave us an approximate measure of how actual pain tolerance differed from participants’ subjective estimate about this tolerance, or, in other terms, how much variance there was in their subjective tolerance estimate in comparison to the objective tolerance. The prospective *posterior belief* was the final prospective pain estimate participants gave after trial 2.

Models 1 and 2 were baseline models, assuming zero (Model 1; prior belief = posterior belief) and 100% (Model 2; posterior belief = evidence) learning. In Model 3 (see Fig. 1, panel b), *prior precision* was approximated by epistemic uncertainty about the *social advice* (i.e., how much participants changed their mind about their pain tolerance after the advice), formally the difference between participants’ pain tolerance estimates before and after the advice (termed “social learning model”). In Models 4 and 5, *prior precision* was approximated by epistemic uncertainty about *sensory learning* sources (i.e., how much participants changed their mind about their pain tolerance after sensory evidence in the previous trial). Model 4 used as *prior precision* the difference between the very first prospective pain tolerance estimate and the prospective estimate following trial 1, and was termed “prospective embodied learning model” as it focused on prospective estimates prior to the social advice. Model 5 used as *prior precision* the difference between actual pain tolerance on trial 1 and the pain tolerance guess on trial 1 and was termed “retrospective embodied learning model” because it focused on measures pertaining to pain experience in trial 1. Full model specifications are presented in the Supplementary Materials (Table S1).

##### 2.5.2.1 Evaluating model fit in terms of correspondence between computed and actual posterior beliefs

To understand how people form beliefs based on various sources of subjective uncertainty, we examined the fit of all five models in terms of how posterior beliefs generated by the computational model corresponded with participants’ actual posterior beliefs. We ran simulations of all possible values (uniform between 0.01 and 10) for free model parameter scale factor *s* (one global value across participants, for each condition and model) and found the value of *s* which optimised the fit of each model. To compare between models, the parameter *s* which had the best fit in each model was used. The Bayesian Information Criterion (BIC) was used to compare Models 3–5 based on how well they predicted the prospective posterior belief. Differences in model fit (absolute error and squared error comparisons) between the social learning and the two embodied learning models were examined using paired-samples *t*-tests.

##### 2.5.2.2 Comparing learning rates between trustworthiness conditions

To explore whether participants showed greater learning under low- vs. high-trustworthiness conditions, we used precision-computed learning rates and compared these between trustworthiness conditions. Specifically, we compared (using Welch 2-sample *t*-test) the learning rates (computed per participant as $\frac{\;Evidence\;Precision\;*s}{\;Evidence\;Precision*s+Prior\;Precision\;}$) of the best model in the low-trustworthiness condition to those of the best model in the high-trustworthiness condition (we also examined actual learning rates; see Supplementary Materials and Table S2). To understand the source of the difference between the learning rates of the two trustworthiness conditions, we then compared the component variables in the learning rates formula, that is, the *evidence precision* and the *prior precision*, between conditions, again using Welch 2-sample *t*-tests.

## 3 Results

### 3.1 Manipulation checks

The two-sample *t*-test revealed that the confederate was reliably perceived to be more trustworthy and positive from the previous feedback in the high-trustworthiness condition (*M* = 6.25, *SD* = 0.72) than in the low-trustworthiness condition (*M* = 2.76, *SD* = 0.96), *t*(68) = −17.35, *p* < .001, *d* = −4.15. Thus, the trustworthiness manipulation was successful.

### 3.2 Descriptive statistics

Due to missing data on some of the covariates, the analyses were conducted on *N* = 63 out of 72 participants (*n* = 29 in the low-trustworthiness condition, *n* = 34 in the high-trustworthiness condition). Descriptive statistics for the outcome variables are presented in Table 1, and correlations between the measures are presented in Supplementary Materials (Table S3).

As expected, participants in the high-trustworthiness condition adjusted their expectation more in the direction of the confederate’s expectation than did those in the low-trustworthiness condition. The advice-taking score of 50.72% in the high-trustworthiness condition indicates that participants gave their own prospective expectation and the confederate’s advice about equal weighting. By contrast, participants in the low-trustworthiness condition only adjusted their expectation by about a third in the direction of the confederate’s advice. Inferential statistics supporting the impact of trustworthiness on advice taking are presented in Section 3.3.

### 3.3 Effects of perceived trustworthiness on advice taking and pain tolerance

**Fig. 2:**
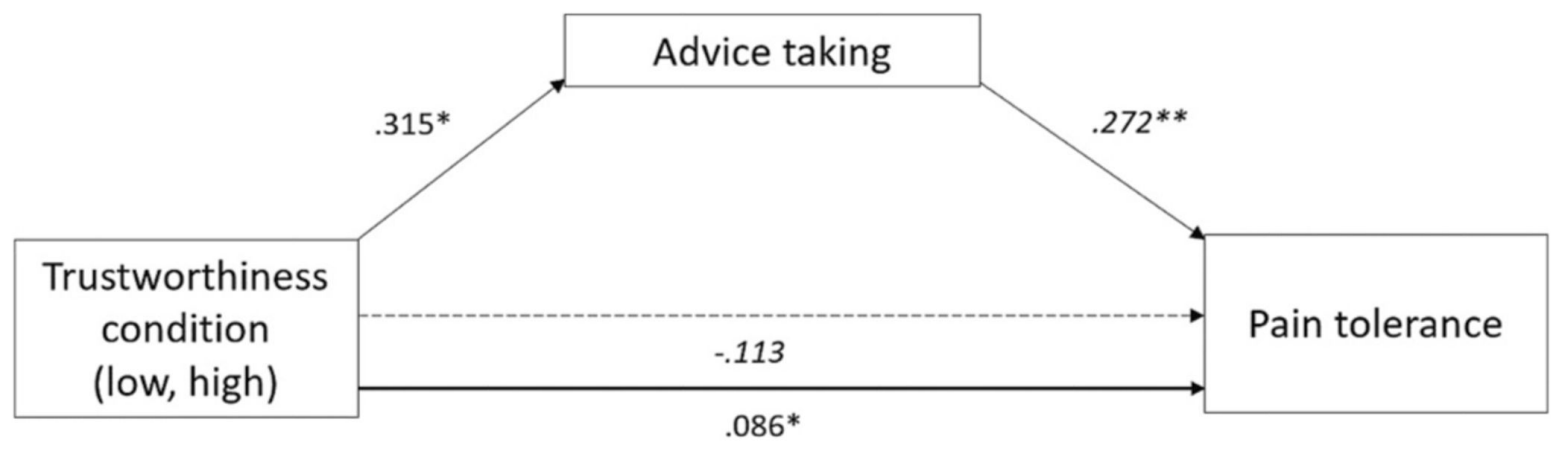
Path diagram showing the paths from trustworthiness condition to advice taking, advice taking to pain tolerance, and trustworthiness condition to pain tolerance on trial 2. Standardised coefficients are shown (see Table 2 for unstandardised coefficients). The dotted line denotes a non-significant direct path, the bold line denotes the indirect effect of trustworthiness condition on pain tolerance via advice taking. * = *p* < .05, ** = *p* < .01.

To assess whether trustworthiness influenced advice taking, and whether advice taking impacted pain tolerance, we specified a path model with direct paths from trustworthiness condition to advice taking and pain tolerance, from advice taking to pain tolerance, and the indirect path from trustworthiness condition to pain tolerance via advice taking. The specified path model fit the data very well, χ2(5) = 2.021, *p* = .846; RMSEA = 0.00; CFI = 1.00; SRMR = 0.03 (see Fig. 2 for the path diagram).

**Fig. 3:**
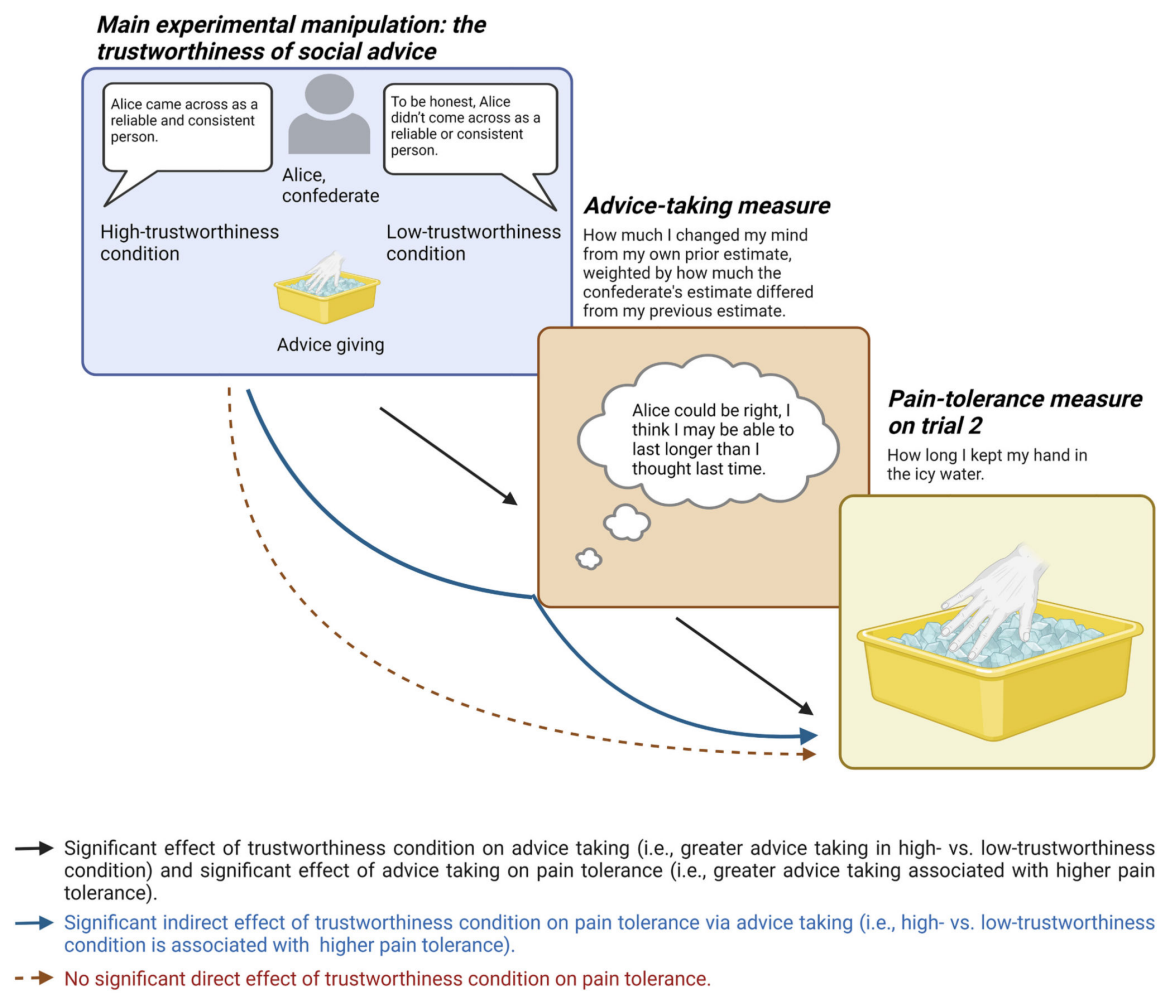
Graphical representation of our path modelling results, showing the effects of trustworthiness condition on pain tolerance via advice taking. (For interpretation of the references to colour in this figure legend, the reader is referred to the web version of this article.)

The direct path from trustworthiness condition to advice taking was significant (see Table 2 for all model results). Participants in the high-trustworthiness condition took the confederate’s advice regarding their own pain tolerance more than did those in the low-trustworthiness condition, supporting Hypothesis 1. Furthermore, the direct path from advice taking to pain tolerance was also significant, supporting Hypothesis 2. The more participants took the confederate’s advice, the longer they immersed their hand in the cold water. Trustworthiness condition did not directly predict pain tolerance. Instead, the indirect path from trustworthiness condition to pain tolerance via advice taking was significant. The perceived trustworthiness of the confederate influenced the degree to which participants took on board the confederate’s advice (i.e., how much they adjusted their own expectation to be in line with the confederate’s), which in turn predicted participants’ pain tolerance during trial 2 (see Fig. 3 for a graphical summary). While this does not necessarily imply a causal mechanism, the path via social advice taking explained more of the variance than the direct path that did not take social advice taking into account.

### 3.4 Updating beliefs about pain tolerance under advice taking in the context of varying epistemic uncertainty (different trustworthiness conditions)

In these analyses, we examined the updating of pain tolerance beliefs and specifically assessed the impact of advice taking and trustworthiness condition on the precision of own prior beliefs vs. embodied evidence. In addition to two baseline models (assuming no learning and 100% learning, respectively), we specified three models in which only *prior precisions* were varied (see *Plan of Analysis*). We examined their fit in terms of how generated posterior beliefs corresponded with participants’ actual posterior beliefs, and compared learning rates between trustworthiness conditions to see whether participants showed greater learning under low- vs. high-trustworthiness conditions.

**Fig. 4:**
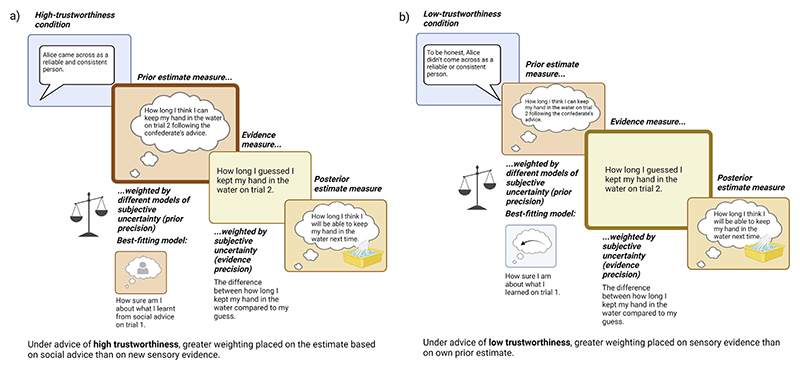
A graphical representation of our computational approximation of belief updating results. Findings are displayed by trustworthiness condition: Greater weighting was placed on the new sensory evidence over one’s own prior estimate in the low-trustworthiness condition (a), while greater weighting was placed on the estimate based on social advice in the high-trustworthiness condition (b). (For interpretation of the references to colour in this figure legend, the reader is referred to the web version of this article.)

Bayesian Information Criterion (lower = better fit) for each model is presented in Table 3. In the low-trustworthiness condition, the retrospective embodied learning model had the best overall fit (specifying prior precision as the difference between actual pain tolerance and guessed pain tolerance on trial 1). In the high-trustworthiness condition, the social learning model had the best overall fit (specifying prior precision as the difference between participants’ tolerance estimates given just prior to and following the advice). The recovered free parameters *s* per model and condition are listed in the Supplementary Materials. Free parameter *s* had a value of 2.61 in the winning model of the high-trustworthiness condition and 1.94 in the winning model of the low-trustworthiness condition (see Supplementary Table S4). The differences in fit (absolute error and squared error comparisons) between the three models varying prior precision were only significant when comparing in the low-trustworthiness condition the social learning model versus the winning retrospective embodied learning model (see Table 3). A graphical representation of these findings is presented in Fig. 4.

**Fig. 5:**
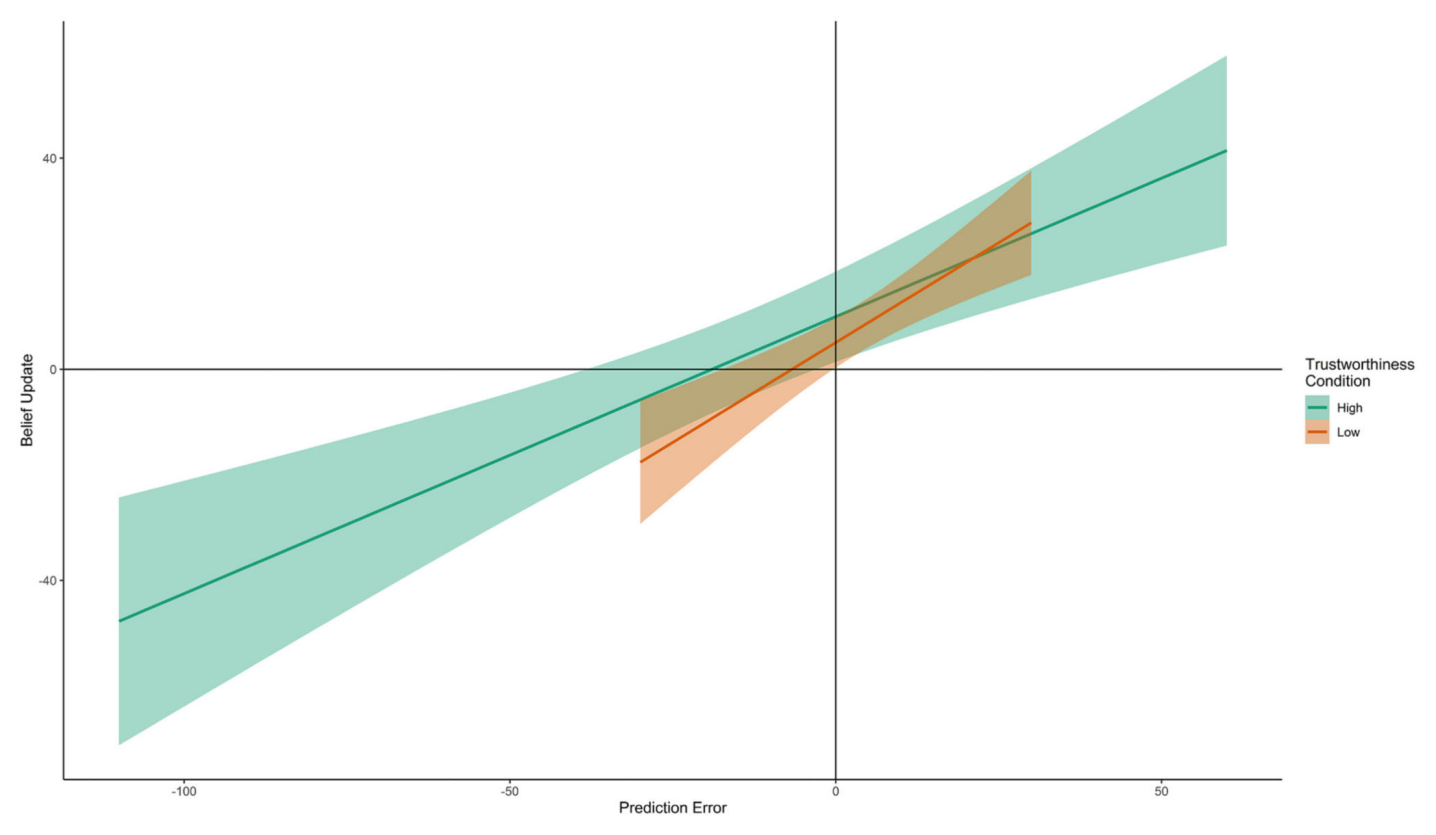
Overall learning rates (slopes) in both trustworthiness conditions, considering that in the Bayesian belief updating framework, the learning rate is the ratio of the belief update (y axis) over the prediction error (x axis). A steeper slope in a condition therefore indicates a higher learning rate across participants in this condition.

In terms of learning rate, the Bayesian precision-based learning rate calculations showed that, as a trend, the learning rate in the low-trustworthiness condition was higher than the learning rate in the high- trustworthiness condition (Welch 2-sample *t*-test, *t*(38.74) = −1.88, *p* = .068; mean difference high vs. low = −0.05; see Fig. 5). Results using actual learning rate, which replicated this finding, are presented in the Supplementary Materials. When we compared the component precisions of the learning rate formula between trustworthiness conditions to explain why the model learning rate was higher, as a trend, for the low-trustworthiness condition than the high-trustworthiness condition, we found statistically significant differences between trustworthiness conditions for *prior precision* (Welch 2-sample *t*-test, *t*(59.10) = 2.64, *p* = .011, mean difference high vs. low = 12.86), but not *evidence precision* (Welch 2-sample t-test, *t*(55.84) = 0.99, *p* = .324, mean difference high vs. low = 12.66). Note that before performing the *t-*test, *evidence precision* proxy values were first multiplied by the relevant condition and model-specific factors *s* to be consistent with the learning rate calculations.

These results support Hypothesis 3: Participants learned more (changed their beliefs more) from new embodied evidence following social advice from a low- (vs. high-) trustworthy source, and the trend towards a higher learning rate in the low- (vs. high-) trustworthiness condition was explained by the fact that prior precision was smaller in the low- vs. high-trustworthiness condition. In other terms, as expected, under conditions where advice came from a person low in trustworthiness, participants had low subjective certainty about their own priors and greater subjective certainty about the new embodied evidence. By contrast, when advice was provided by a highly trustworthy person, participants had greater subjective certainty in their own socially-influenced priors relative to new sensory information.

## 4 Discussion

In this study, we examined the influence of social expectations in the form of explicitly communicated, personalised social advice regarding participants’ likely pain tolerance and the perceived trustworthiness of the person expressing the expectation on advice taking, pain tolerance, and updating beliefs about pain tolerance. We drew on Bayesian learning conceptualisations that individuals strive to reduce the discrepancy between their own and social expectations to minimise the uncertainty of their environment (Theriault et al., 2021) and extended this view to consider the conditions under which advice taking would be most likely to occur. In Bayesian brain frameworks, higher epistemic trust should confer greater subjective certainty, in the sense of epistemic unambiguity, about social expectations, and thus increase their weighting in shaping one’s own expectations relative to new sensory evidence. Therefore, we expected that when receiving explicit advice, participants would adjust their pain expectations to match the expressed expectations more when provided by a high- vs. low-trustworthy adviser. We further expected that the degree to which participants took on board advice would predict their subsequent pain tolerance. Finally, we expected that in updating beliefs about pain tolerance, greater precision would be conferred to socially-influenced prior beliefs rather than sensory evidence in the high-trustworthiness condition, with opposite effects in the low-trustworthiness condition.

Our results confirmed these hypotheses. We found that, as expected, perceived trustworthiness influenced advice taking, with participants adjusting more to the advice in the high- vs. low-trustworthiness condition. This finding is in accordance with results from economic trust games, in which participants were more likely to take the advice of honest vs. dishonest advisers (Bellucci, Molter, & Park, 2019). We also found direct effects of social advice taking on pain tolerance. This finding extends literature using, for example, social observational learning paradigms, to demonstrate that the degree to which participants take on board personalised advice during a live social interaction (which in turn depends on the trustworthiness of the advice giver) predicts their subsequent pain tolerance. Studies have shown that trustworthiness facets such as warmth and competence (see Colquitt et al., 2007) influence pain expectations (see e.g., Blasini et al., 2018; Howe et al., 2017; Necka et al., 2021), pain (Campbell et al., 2006), and neural activation such as the neurologic pain signature, which is specific to pain (Anderson et al., 2023). In the present study, trustworthiness did not predict pain tolerance directly, but rather, the indirect path from perceived trustworthiness to pain tolerance via social advice taking was significant. Together, these findings indicate that social sources varying in perceived trustworthiness can influence not only how we experience the external world, but also how we experience signals originating in our own body, through influencing our own expectations about pain.

Moreover, we showed how advice delivered by a highly trustworthy adviser affected pain tolerance by influencing not only the content of pain expectations, as our behavioural results show, but also the subjective certainty by which they were held, thus influencing the malleability of one’s beliefs about pain by new sensory evidence. Specifically, under conditions of advice from a low-trustworthy adviser, participants had low subjective certainty about their own prior beliefs, as influenced by the confederate, and greater subjective certainty about the new embodied evidence. In this condition of high epistemic ambiguity, participants tended to mistrust their own (socially-influenced) beliefs more and be more liable to changing them based on new, in this case sensory, evidence, while the opposite applied to conditions of advice by a highly trustworthy adviser. The latter seemed to influence prior beliefs and increase their precision relative to the precision of new sensory evidence, so that relatively less learning was achieved by new sensory evidence. The trend towards a greater learning rate in the low-trustworthiness condition is congruent with Bellucci et al. (2019)’s findings that participants adjusted their behaviour during a trust game more quickly in low- vs. high-honesty conditions. This might be because there is more at stake – low trustworthiness is threatening (Oosterhof & Todorov, 2008) and characterised by epistemic uncertainty. Our study suggests that in such circumstances, we may need to lend more weight to our embodied experience to reduce uncertainty over allowing socially-influenced expectations to shape our expectations.

Our paper demonstrates the importance of social context, and in particular social expectations, in modulating pain. In previous theoretical work, we have indeed argued that the development of bodily and particularly interoceptive priors in humans is shaped by social interactions, especially in early life (Fotopoulou et al., 2022; Fotopoulou & Tsakiris, 2017). Human infants are born with very limited possibilities for purposeful action and interoceptive self-regulation, such as independent thermoregulation, nourishment, or danger avoidance. Instead, they show a strong social orientation and reflexes for eliciting social attention (e.g., the crying reflex) and rely on their proximal, social affiliations for the regulation of their interoceptive states. Hence, in the early years of complete dependency on embodied caregiving social interactions (Fotopoulou et al., 2022; Fotopoulou & Tsakiris, 2017), and perhaps even already in utero (Ciaunica, Constant, Preissl, & Fotopoulou, 2021), the interoceptive experiences of infants, and hence the formation of their interoceptive priors, are directly influenced by their caregivers. For example, it is the caregiver that needs to interpret and respond to the needs of a crying infant, both in terms of deciding whether the infant is hungry, cold, tired, or in pain, and in terms of how and when to regulate such needs, or at least how to try to do so and then observe the infant’s response, at times naming it, and also soothing it. Thus, as with other aspects of interoception, caregivers are necessary for enacting and explicitly teaching babies about pain and the necessary responses to it, including both embodied and cognitive responses. Our early experiences of pain are therefore fundamentally intersubjective in nature, and there is evidence that intersubjective social, and cultural expectations continue to influence our own pain expectations and experiences in adulthood (Che, Robin, Sungwook, Paul, & Bernadette, 2018; Krahé et al., 2013; Krahé & Fotopoulou, 2018). It has also been shown that children learn more about the world from reliable adults that yield greater epistemic trust than unreliable ones (Clément, Koenig, & Harris, 2004; Koenig & Harris, 2007). In this paper, we show similar processes in adulthood and specifically as regards interoceptive and epistemically-private experiences, namely pain.

To our knowledge, our empirical insights regarding the dynamic balance between social advice, own expectations, and sensory evidence are unprecedented in the literature and can lead to novel hypotheses. For example, the increased bodily focus seen in chronic pain conditions, as well as in many other conditions at the physical-mental health interface such as somatic symptoms, functional and eating disorders, may be related to active, yet untrustworthy sources of social support in current life or in the course of development. Whether or not others are generally seen as untrustworthy is shaped by individuals’ attachment styles, that is, cognitive schemata around the availability of others to one’s needs in times of threat. Unreliable or unresponsive caregiving in early life is thought to lead to the development of insecure (anxious and avoidant) attachment styles, characterised by low trust in others (Mikulincer, 1998). Indeed, in adulthood, greater attachment insecurity moderates effects of social context on pain experience, such that, for example, experiencing pain in the presence (vs. absence) of one’s partner is associated with greater pain in the context of higher attachment avoidance (Krahé et al., 2015). We have previously hypothesised that the presence of a partner is at odds with more avoidantly attached individuals’ preferred strategy to cope with threat on their own, thus maintaining rather than reducing the salience of noxious stimuli (Krahé et al., 2015). In this vein, future research could consider the role of individual differences in attachment style in weighting embodied experience vs. socially-influenced expectations in belief updating about pain in a social context.

Furthermore, information about a person’s trustworthiness can be acquired by direct interaction or indirectly through reputation (Terenzi, Liu, Bellucci, & Park, 2021). In this study, we combined both by first showing participants (false) feedback from previous participants and then asking them to complete a baseline trial during which they acquired first-hand experience of the confederate. While individuals make trustworthiness judgments on the basis of facial expressions (Todorov, Baron, & Oosterhof, 2008), we presented the trustworthiness manipulation before participants met the confederate, and the same confederate was used in the low- and high-trustworthiness conditions, keeping facial features constant. Moreover, we drew on different facets of trustworthiness to create a global trustworthiness manipulation, rather than focusing on e.g., competence or integrity on their own. While examining sub-facets of trustworthiness is important, research shows that perceived trustworthiness is a more useful global indicator when forming general impressions of others than are warmth and competence per se (e.g., Brambilla, Rusconi, Sacchi, & Cherubini, 2011). The ecological validity of our trustworthiness manipulation was a strength of this study. Previous studies have used facial expressions (e.g., Slepian et al., 2012) and vignettes (e.g., King, Rowe, & Leonards, 2011) to manipulate trustworthiness. However, we chose a manipulation which was related to evaluating the confederate’s role in a face-to-face interaction with possibility for action (see Krahé et al., 2013), similar to interactions taking place in medical contexts. By posing the confederate as a medical student, who also monitored the coldpressor during the trial, we aimed to make the trustworthiness manipulation relevant to the pain experience. The trustworthiness manipulation was carefully developed and piloted, and perceived by participants in the intended manner, supporting its validity. In Bayesian terms, social epistemic actions, such as the social advice provided by the confederate in this study, are ‘quintessentially communicative’ (Pezzulo, Barca, Maisto, & Donnarumma, 2020). Exploring their impact within ecologically-valid social interactions, rather than in implied, abstract ways, thus seems imperative.

Furthermore, entering our variables into the path model in the temporal order in which they were measured allowed us to examine the influence of perceived trustworthiness and social advice taking on subsequent pain. A limitation of the study was that, due to using the coldpressor apparatus and cooling the hand substantially during the trial, we were limited to two trials (one per hand; baseline and main trial). Future studies could use other established pain paradigms (e.g., heat pain, as in Koban & Wager, 2016) affording more pain trials and opportunities to see changes in pain expectations unfold dynamically over time, although the ecological validity of such experiments is typically limited. Furthermore, our sample was all female, and so we were unable to test sex differences or interactions between the sex of the participant and the sex of the person giving social advice on our outcomes. As our confederate was female, we wanted to hold sex constant and recruited only female participants into the study. We also considered that including a male confederate, although allowing us to recruit male participants, would raise concerns around the comparability of the male and female confederates in terms of trustworthiness, as e.g., facial features have been linked to trustworthiness (Oosterhof & Todorov, 2008) and effects on pain (Mattarozzi et al., 2020), as outlined above. Therefore, we decided to keep the confederate constant and vary perceived trustworthiness only for this one person. However, it is important for future research to explore whether our results would be replicated in male participants, and for different sexes in both the confederate and participant role. In addition, the confederate’s expectation of participants’ pain tolerance was designed to always be higher than participants’ own expectation, and participants who reached the maximum immersion time were told to withdraw their hand, rather than choosing when to withdraw their hand themselves, which may have made a difference. Future studies could examine whether a highly trustworthy confederate providing expectations which are lower than participants’ expectations (e.g., of reduced pain tolerance) would lead participants to adjust their own expectations of pain tolerance downwards, as well as more generally investigate changes in trustworthiness and social expectations along a continuum. In this vein, explicitly testing effects of congruence vs. incongruence in own vs. social expectations, and how especially incongruence impacts subsequent pain, is a question for future research (see also Schrooten & Linton, 2017). Lastly, while we focused on trustworthiness, factors such as whether a social expectation is communicated by an in-group or out-group member may also modulate precision of such expectations (Albarracin et al., 2022).

In the present study, we focused on the perceived trustworthiness of a person communicating their expectations about participants’ pain to participants. While we have previously argued that social factors may modulate pain by clearly and unambiguously signalling reduced threat/relative safety of incoming sensory information or safety of the environment in which it occurs (see e.g., Krahé et al., 2013; Krahé & Fotopoulou, 2018), we here did not directly test expectations about safety but rather expectations about pain tolerance and experience, that is, active and perceptual inferences about pain itself. While it is possible that the highly trustworthy person ostensibly in charge of participants’ safety during a coldpressor task, who then advised participants that they would tolerate the coldpressor for longer than they themselves thought, might have also influenced expectations regarding bodily safety, future studies would need to more directly manipulate expectations around stimulus safety (see e.g., Wiech et al., 2010) to draw conclusions about the impact of perceived trustworthiness on expectations of safety and threat.

Moreover, social interactions are dynamic and reciprocal in nature, and examining characteristics of the person in pain seems an important avenue for future research. For example, empathic responses are greatly reduced when a person in pain appears low vs. high in trustworthiness (Sessa & Meconi, 2015). In Sessa and Meconi (2015)‘s study, trustworthiness was manipulated by facial appearance, and indeed others have found that judgments of patients’ pain are influenced by such superficial features (Ashton-James & Nicholas, 2016). Such judgments may feed into expectations which are communicated to the person in pain, and can in turn affect pain outcomes. As well as studying features of the person in pain, measuring personality traits related to perceiving others as high or low trustworthy and trusting others, such as individual differences in attachment style mentioned above, and epistemic trust, could yield more fine-grained results regarding when and for whom the communication of social expectations in relation to pain may have a beneficial or detrimental effect. Moreover, we focused here on an interpersonal interaction, and therefore chose to use the term ‘advice taking’ over related concepts such as ‘conformity’, which also denotes yielding to social pressure or adapting one’s expectations to match others’ expectations, but often in group or intergroup situations. We did not capture these group or majority dynamics here; however, previous work has indeed highlighted the differences between some of the wider social motivations that may underlie social conformity (such as reputational costs or specific social sanctions) vs. the more inter- or intra-personal motivators for taking on someone’s explicit advice, or following their assumed expectations (such as the potential felt, moral ‘sense of should’; Theriault et al., 2021).

Our findings have implications for healthcare settings, in which doctors may communicate their expectations regarding pain to patients. Fostering positive practitioner-patient relationships, in which the practitioner is seen as a trustworthy social partner, may be especially beneficial for influencing positive pain expectations (see Blasini et al., 2018). Interestingly, while participants in the high-trustworthiness condition took the social expectation into account, they also gave their own expectation relatively equal weighting (see advice taking results). Thus, social expectations from a highly trustworthy other did not override own expectations – indeed, this would be surprising given that participants did have the experience of the previous baseline trial to draw on in forming their expectation. However, the social expectation increased the precision of participants’ prior beliefs relative to the precision of new sensory evidence, so that less learning was achieved by new sensory evidence. In conditions of low trustworthiness, people may instead rely on the precision of own embodied experience in updating beliefs.

In conclusion, we found that effects of personalised, explicit social expectations on own pain beliefs and pain tolerance depend on the perceived trustworthiness of the person providing the social expectation. It thus seems important to enhance positive perceptions of individuals communicating expectations about pain, e.g., in doctor-patient interactions, to lead to more positive pain outcomes.

## Supplementary Material

**Appendix A. Supplementary data**

Supplementary data to this article can be found online at https://doi.org/10.1016/j.cognition.2024.105756.

Supplementary material

## Figures and Tables

**Table 1:** Descriptive statistics by trustworthiness conditions (low, high).

					Low				High			
					Mean		SD		Mean		SD	
	Trial 1		PE1		80.21		30.85		75.44		26.27	
			Pain threshold		19.55		13.59		23.17		16.83	
			First pain intensity rating		47.89		23.04		44.26		28.78	
			Final pain intensity rating		85.41		20.36		85.18		21.82	
			Pain tolerance		109.59		63.21		105.77		62.81	
			Retrospective pain estimate		69.72		50.16		61.21		36.29	
			PE2		66.66		37.54		66.03		43.59	
	Advice taking				36.18		29.27		50.72		36.22	
	Trial 2		PE3		81.48		52.49		81.97		54.11	
			Pain threshold		18.10		11.97		21.38		21.53	
			First pain intensity rating		52.90		24.87		45.82		31.50	
			Final pain intensity rating		83.45		20.21		81.94		21.30	
			Pain tolerance		115.53		64.58		116.89		63.47	
			Retrospective pain estimate		84.52		56.24		70.29		45.06	
			PE4		88.86		59.85		85.82		58.04	

**Table 2:** Full path analysis results displaying unstandardised coefficients.

	Outcome variable	Predictor variable	*b*	*S.E.*	*p*	95% CI		
						Lower	Upper	
		Trustworthiness condition	21.19	8.72	0.015	6.59	35.09	
	Advice taking	Hand temperature*	–3.80	3.79	0.315	–9.36	2.99	
		Age*	2.02	0.89	0.024	0.71	3.60	
		Trustworthiness condition	–14.39	13.05	0.270	–35.14	7.54	
Direct effects		Advice taking	0.51	0.17	0.002	0.23	0.79	
	Pain tolerance	Pain threshold*	0.36	0.50	0.477	–0.52	1.11	
		First pain intensity rating*	–0.79	0.30	0.009	–1.30	–0.30	
		Final pain intensity rating*	–0.99	0.26	0.000	–1.44	–0.59	
	Intercepts	Advice taking	96.55	112.13	0.389	–104.98	262.13	
		Pain tolerance	215.37	33.88	0.000	163.77	274.54	
Indirect effects	Pain tolerance	Trustworthiness condition via advice taking	10.90	5.52	0.048	2.44	20.30	

Note. * = covariates.

**Table 3:** Bayesian Information Criterion (BIC) and model comparisons by high- and low-trustworthiness condition.

			Low-trustworthiness condition (*n* = 29)			High-trustworthiness condition (*n* = 33)		
			BIC	Paired *t-*test on absolute error of predictions		BIC	Paired *t-*test on absolute error of predictions	
	Baseline: 0% learning		249.8			327.3		
	Baseline: 100% learning		236.7			324.8		
	Social learning		234.0	social learning vs. embodied learning (prospective): *t*(28) = 1.02, *p *= .315, mean difference = 0.25	* *	*301.2*	*social learning* vs. *embodied learning (prospective): t(32) = –0.72, p* *= .476, mean difference = –1.50*	* *
	Embodied learning (prospective)		232.9	social learning vs. embodied learning (retrospective): *t*(28) = 2.25, *p *= .033, mean difference = 0.44		317.9	social learning vs. embodied learning (retrospective): *t(*32) = 0.55, *p *= .583, mean difference = 0.26	
	Embodied learning (retrospective)	* *	*232.2*	*embodied learning (prospective) vs. embodied learning* *(retrospective): t(28) = 0.89, p = .382, mean difference = 0.19*		302.8	embodied learning (prospective) vs. embodied learning (retrospective): *t(*32) = 0.92, *p *= .366, mean difference = 1.75	

*Note*. Best-fitting model highlighted in italics in each trustworthiness condition.

## Data Availability

Data will be made available on request.
