# Supplementary material for: Updating beliefs about pain following advice: Trustworthiness of social advice predicts pain expectations and experience
Source: Cognition. Author manuscript; Available in PMC 2024 Nov 1. (PMC7616089; doi:10.1016/j.cognition.2024.105756)
Supplement: Supplementary material [file EMS195725-supplement-Supplementary_material.docx]

**SUPPLEMENTARY MATERIALS**

**Statements used for the trustworthiness manipulation**

*High-trustworthiness condition*:

1. “Alice came across as a reliable and consistent person.” (Integrity, *M* = 6.00, *SD* = .88)
2. “I noticed that Alice really paid attention to me to try and give an accurate estimate and keep me safe.” (Benevolence, *M* = 6.00, *SD* = 1.31)
3. “I got the impression very quickly that Alice was a caring person and keen to estimate my pain correctly and safely.” (Benevolence, *M* = 5.93, *SD* = 1.28)
4. “I noticed that Alice stayed focused and attentive to my pain throughout the experiment.” (Integrity, *M* = 5.88, *SD* = .93)

*Low-trustworthiness condition*:

1. “To be honest, Alice didn’t come across as a reliable or consistent person.” (Integrity, *M* = 1.60, *SD* = 1.30)
2. “I didn’t find Alice particularly caring and wasn’t sure whether her estimate was safe.” (Benevolence, *M* = 1.60, *SD* = .91)
3. “I noticed that Alice didn’t seem to pay attention to the first pain trial which would have been necessary to give an accurate estimate and make me feel safer.” (Benevolence, *M* = 1.88, *SD* = 1.22)
4. “Alice didn’t seem that interested in becoming good at estimating others’ pain. It’s a shame because it could help her be a good and caring doctor.” (Integrity, *M* = 2.11, *SD* = 1.15)

**Bayesian belief updating framework**

Within the Bayesian belief updating framework, posterior beliefs are “generated” as the precision-weighted average of prior beliefs and evidence:

$$Posterior=Prior+\frac{Precision of Evidence}{Precision of Evidence+Precision of Prior}(Evidence-Prior)$$

In our experiment, we did not have enough trials to allow us to calculate actual precisions for evidence and prior; therefore, we used proxies for our five models, defined in the “Precision of Evidence” and “Precision of Prior” columns in Table S1.

## Table S1. *Model descriptions.*

| **Model Name** | **Prior** | **Evidence** | **Posterior** | **Precision of Evidence** | **Precision of Prior** |
| --- | --- | --- | --- | --- | --- |
| Baseline: No Learning | Pain estimate 3 | Pain tolerance guess 2 | Pain estimate 4 | 0 | 1 |
| Baseline: 100% Learning | Pain estimate 3 | Pain tolerance guess 2 | Pain estimate 4 | 1 | 0 |
| Social Learning | Pain estimate 3 | Pain tolerance guess 2 | Pain estimate 4 | (Pain tolerance 2 - Pain tolerance guess 2)/Pain tolerance 2 | (Pain estimate 2 - Pain estimate 3)/Pain estimate 2 |
| Prospective embodied learning | Pain estimate 3 | Pain tolerance guess 2 | Pain estimate 4 | (Pain tolerance 2 - Pain tolerance guess 2)/Pain tolerance 2 | (Pain estimate 1 - Pain estimate 2)/Pain estimate 1 |
| Retrospective embodied learning | Pain estimate 3 | Pain tolerance guess 2 | Pain estimate 4 | (Pain tolerance 2 - Pain tolerance guess 2)/Pain tolerance 2 | (Pain tolerance 1 - Pain tolerance guess 1)/Pain tolerance 1 |

*Note*. “Baseline: No Learning” assumes no learning, in which case the posterior is the same as the prior. “Baseline: 100% Learning” assumes 100% learning, in which case the posterior is the same as the evidence.

Considering we used precision proxies, and not precisions of actual probability distributions, we added a scale factor *s* because we needed the models to capture how the relative changes of $\pi_{\vartheta}$ reflected participants’ belief updating, rather than $\pi_{\vartheta}$ proxy absolute level:

$$Posterior=Prior+\frac{Precision of Evidence*s}{Precision of Evidence*s+Precision of Prior}(Evidence-Prior)$$

Scale factor *s* was calculated as a global parameter, across all participants per condition and model, as we did not have enough trials to calculate a factor per participant.

The precision-weighted ratio in the formula above corresponds to the learning rate:

$$Learning Rate=\frac{Precision of Evidence*s}{Precision of Evidence*s+Precision of Prior}$$

**Actual learning rates across trustworthiness conditions**

Drawing on the structural equivalence between the belief updating formula and the linear regression model, we compared actual learning rates between trustworthiness conditions by performing a linear regression with belief update as outcome, and pain tolerance guess on trial 2, prospective pain estimate following confederate advice but before trial 2 (PE3), and the trustworthiness condition by PE3 interaction as fixed effect predictors.

Results from the linear regression analysis (see Table S2) indicated that the actual learning rate in the low-trustworthiness condition was 0.53 (significant) and that the actual learning rate of the high-trustworthiness condition was lower by 0.10 (significant). These results replicated the Bayesian precision-based learning rate calculations presented in the main paper.

Table S2*. Regression analysis on actual learning rate.*

| **Predictors** | **Estimates** | **95% CI** | ***p*** |
| --- | --- | --- | --- |
| Retrospective pain estimate on trial 2 | 0.66 | .49 - .82 | < .001 |
| PE3 | -0.53 | -.71 - -.35 | < .001 |
| trustworthiness condition x PE3 | 0.10 | .00 - .20 | 0.043 |
| Random effects |  |  |  |
| σ^2^ | 258.37 |  |  |
| τ_00_ _hand order_ | 64.26 |  |  |
| τ_00 phrasing_ | 42.51 |  |  |
| ICC | 0.29 |  |  |
| N _hand order_ | 2 |  |  |
| N _phrasing_ | 2 |  |  |
|  |  |  |  |
| Observations | 62 |  |  |
| Marginal R^2^/Conditional R^2^ | 0.439/0.603 | |  |

*Note.* PE2 = Prospective pain estimate 2, provided before the social estimate; PE3 = Prospective pain estimate 3, provided after the social estimate.

Table S3. *Correlations between measures.*

|  | Initial pain tolerance estimate | Updated pain tolerance estimate | Social  update | Pain threshold | First pain intensity rating | Final pain intensity rating | Pain tolerance |
| --- | --- | --- | --- | --- | --- | --- | --- |
| Initial pain tolerance estimate | 1.00 |  |  |  |  |  |  |
| Updated pain tolerance estimate | 0.96** | 1.00 |  |  |  |  |  |
| Social update | 0.13 | 0.34* | 1.00 |  |  |  |  |
| Pain threshold | 0.28* | 0.20 | -0.06 | 1.00 |  |  |  |
| First pain intensity rating | -0.41** | -0.35* | 0.01 | -0.59** | 1.00 |  |  |
| Final pain intensity rating | -0.52** | -0.45** | 0.02 | -0.29* | 0.31* | 1.00 |  |
| Pain tolerance | 0.65** | 0.66** | 0.23 | 0.38* | -0.50** | -0.45** | 1.00 |

Note. * = *p* < .05; ** = *p* < .001.

Table S4. *Model free parameter s (precision of evidence scaling parameter) results.*

|  | *s* ($\pi_{\varepsilon}$ scaling factor) | |
| --- | --- | --- |
| Model Name | Low-trustworthiness condition | High-trustworthiness condition |
| Baseline: No Learning | n/a | n/a |
| Baseline: 100% Learning | n/a | n/a |
| Social Learning | 2.42 | **2.61** |
| Prospective embodied learning | 1.98 | 5.75 |
| Retrospective embodied learning | **1.94** | 1.76 |

We notice in the scaling factors above (the factors of the winning model per condition in bold), that they all have “reasonable” values, with the exception of the retrieved factor in the *prospective embodied learning* model of the *high-trustworthiness* condition, which is clearly too high. This does not impact our findings as this specific model was not the winner of the *high-trustworthiness* condition.
